# Supplementary material for: Epigenetic regulation of the circadian gene Per1 contributes to age-related changes in hippocampal memory
Source: Nat Commun. 2018 Aug 20;9:3323. doi: 10.1038/s41467-018-05868-0 (PMC6102273; doi:10.1038/s41467-018-05868-0)
Supplement: Supplementary file 1 — Supplementary Information [file 41467_2018_5868_MOESM1_ESM.pdf]

Supplementary Information

Epigenetic regulation of the circadian gene *Per1* in the hippocampus contributes to age-related changes in memory and synaptic plasticity

Kwapis et al.

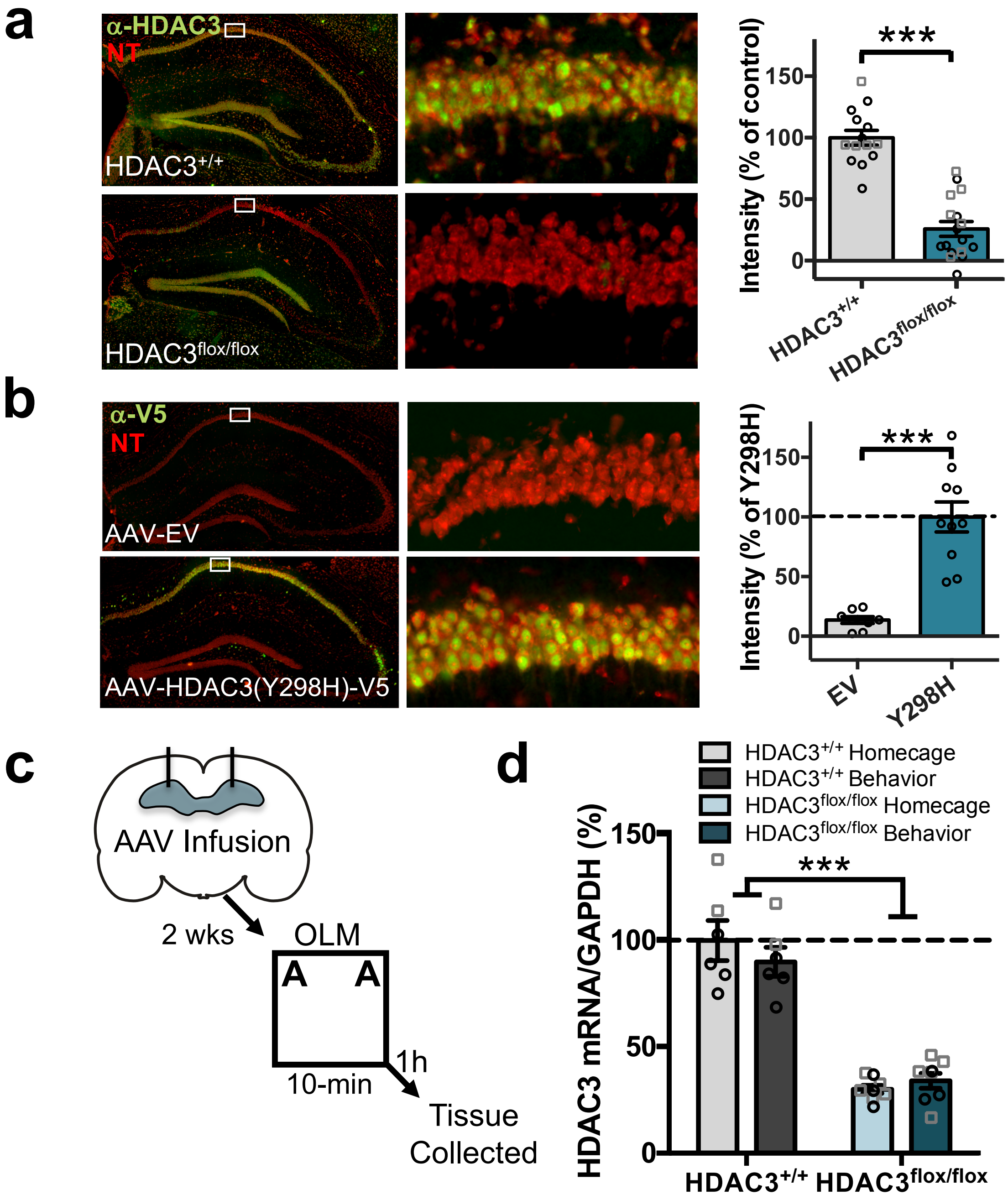

Supplementary Figure 1. Genetic deletion or activity-specific disruption of HDAC3 in the dorsal hippocampus of aging mice. (a) Representative immunofluorescence images of HDAC3 (green) expression in HDAC3$^{+/+}$ and HDAC3$^{flox/flox}$ mice injected with AAV2.1-CaMKII-Cre. Cell bodies were counterstained with a fluorescent Nissl stain (NeuroTrace, red). **Right**: mean intensity of HDAC3 immunofluorescence sampled from CA1 (normalized to background). HDAC3 expression was significantly reduced in HDAC3$^{flox/flox}$ mice compared to HDAC3$^{+/+}$ controls ($t(30)=8.45$, ***$p<0.0001$, $n=14$(5F), 18(6F)). **(b)** Representative immunfluorescence images of V5 (green) expression in wildtype mice injected with AAV-EV (Empty Vector control virus, top) or AAV2.1-CMV-HDAC3(Y298H)-V5 (bottom) counterstained with NeuroTrace (red). The V5 epitope tag added to HDAC3(Y298H)-V5 allowed us to selectively detect the HDAC3(Y298H)-v5 mutant without picking up endogenous HDAC3. **Right**: Mean intensity of V5 immunofluorescence (normalized to background). V5 expression was significantly higher in mice injected with AAV-HDAC3(Y298H)-V5 compared to AAV-EV controls ($t(17)=6.33$, $p<0.0001$, $n=9,10$, all male). **(c)** Experimental procedure. **(d)** HDAC3 mRNA expression in the dorsal hippocampus. HDAC3$^{+/+}$ (gray bars) or HDAC3$^{flox/flox}$ (blue bars) mice infused with AAV-Cre were sacrificed at rest (homecage) or 60m after OLM (OLM). HDAC3 mRNA expression was significantly reduced in HDAC3$^{flox/flox}$ animals compared to HDAC3$^{+/+}$ animals regardless of whether they received OLM training (Genotype group difference only, $F(1,24) = 132.1$,***$p<0.0001$, $n=6$(2F), 6(3F), 8(5F), 8(5F)). Data are presented as mean ±SEM; Black circles, males, gray squares, females.

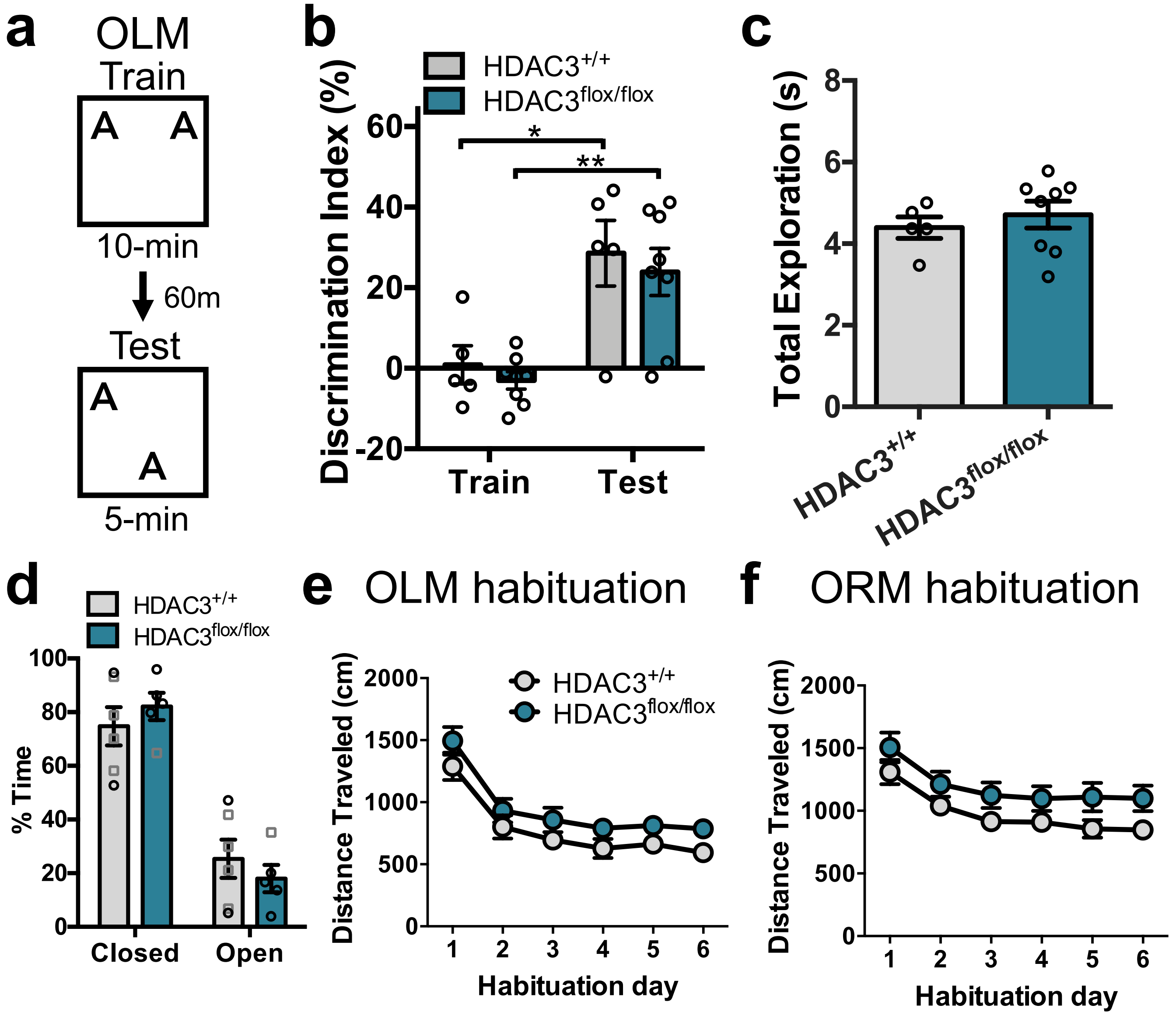

Supplementary Figure 2. All groups show normal short-term memory, movement, and anxiety. (a) Experimental schematic testing short-term memory. Two weeks after AAV-CaMKII-Cre infusion into the DH, 18-month-old HDAC3$^{+/+}$ and HDAC3$^{flox/flox}$ mice were trained in OLM and tested 60m later. **(b)** Both groups showed intact memory for OLM when tested 60m after training (Two-way ANOVA: Main effect of Session (F(1,11)=28.99, *p*<0.001), Sidak's *post hoc* tests, **p*<0.05, ***p*<0.01, no significant differences observed between groups at either training or test, n=5,8; all male). **(c)** Both groups also showed similar levels of total exploration ($t_{(11)}$=0.68, *p*=0.511). **(d)** A subset of animals from Figure 1B were tested in the elevated plus maze. Both groups showed similar levels of anxiety, as indicated by the percentage of time spent in the closed and open arms (Effect of Arm only, $F_{(1,9)}$=38.47, ****p*<0.001, n= 6(4F), 5(1F)). **(e)** Total distance traveled during each day of OLM habituation for mice in Figure 1B. No group differences were observed and individual comparisons between groups each day revealed no significant differences. **(f)** Total distance traveled during ORM habituation for mice in Figure 1G. No group differences were observed and individual comparisons between groups each day revealed no significant differences. Data are presented as mean ±SEM. Black circles, males; gray circles, females.

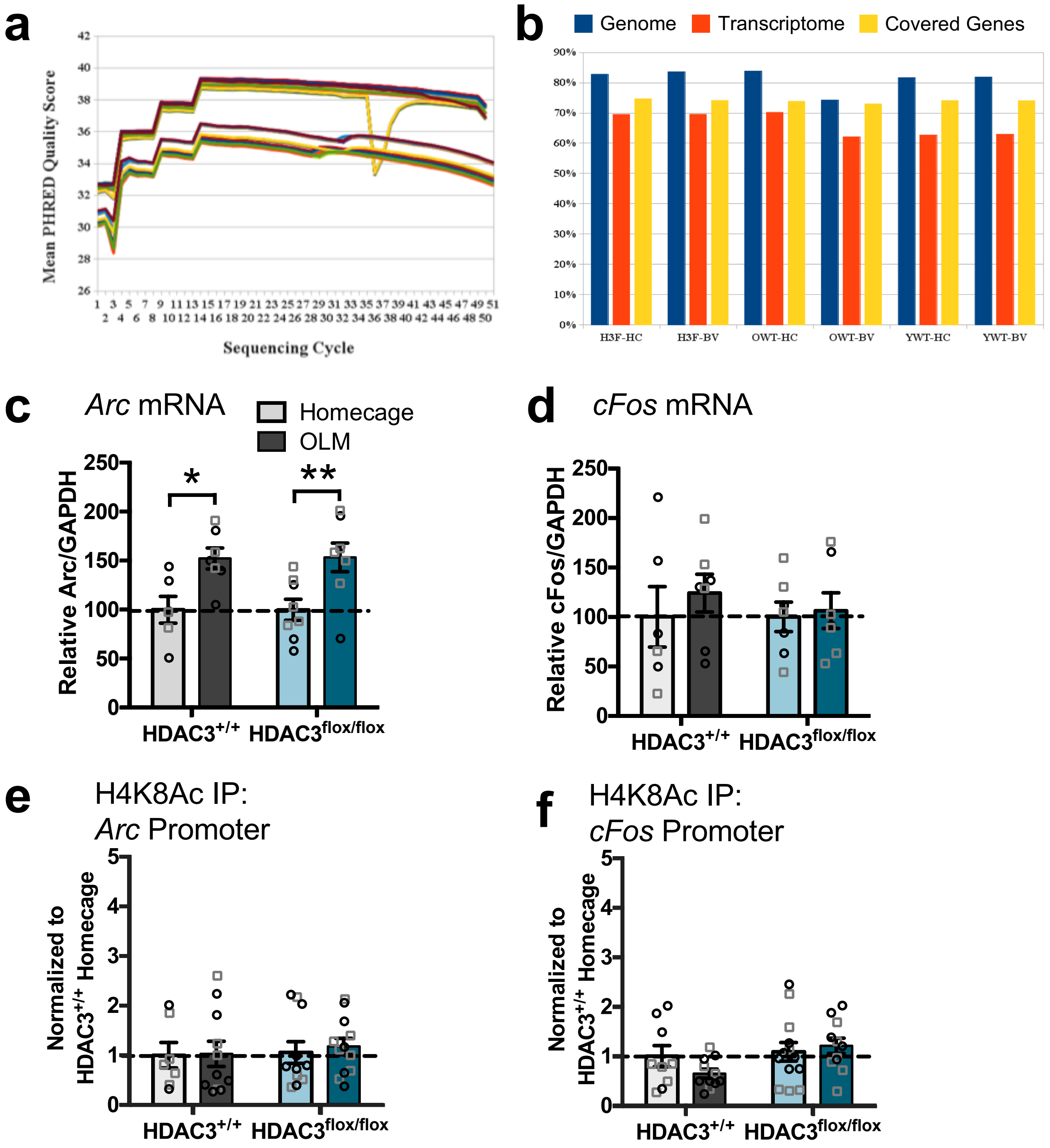

Supplementary Figure 3. RNA sequencing quality controls and characterization of individual genes. (a) Mean PHRED quality scores for each animal in the six different groups: 18-m.o. HDAC3$^{flox/flox}$ homecage (H3F-HC), 18-m.o. HDAC3$^{flox/flox}$ behavior (H3F-BV), 18-m.o. wildtype homecage (OWT-HC), 18-m.o. wildtype behavior (OWT-BV), 3-m.o. wildtype homecage (YWT-HC), and 3-m.o. wildtype behavior (YWT-BV). **(b)** Percentage of short-reads uniquely aligned to the reference genome (blue bars) and transcriptome (red bars) and percentage of annotated genes covered by the sequencing reads (yellow bars) for each of the six groups. **(c-f)** Characterization of individual genes in 18-m.o. wildtype and HDAC3$^{flox/flox}$ mice after learning, from Figure 3. **(c)** *Arc* mRNA expression (Effect of training only ($F_{(1,25)}$=17.31, p = 0.0003), Sidak's *post hoc* tests,*p<0.05, *p<0.01, n=6(2F),7 (3F),8(5F), 8(5F)). **(d)** *cFos* mRNA expression (No significant effects, n=6(2F), 7(3F), 7(4F), 7(5F)). **(e)** H4K8Ac occupancy at the *Arc* promoter (No significant effects, n = 7(5F), 11(4F), 11(4F), 12(7F)). **(f)** H4K8Ac occupancy at the *cFos* promoter (No significant effects, n=9(5F), 11(4F), 13(6F), 11(6F)). Data are presented as mean ±SEM. Black circles, males; gray circles, females.

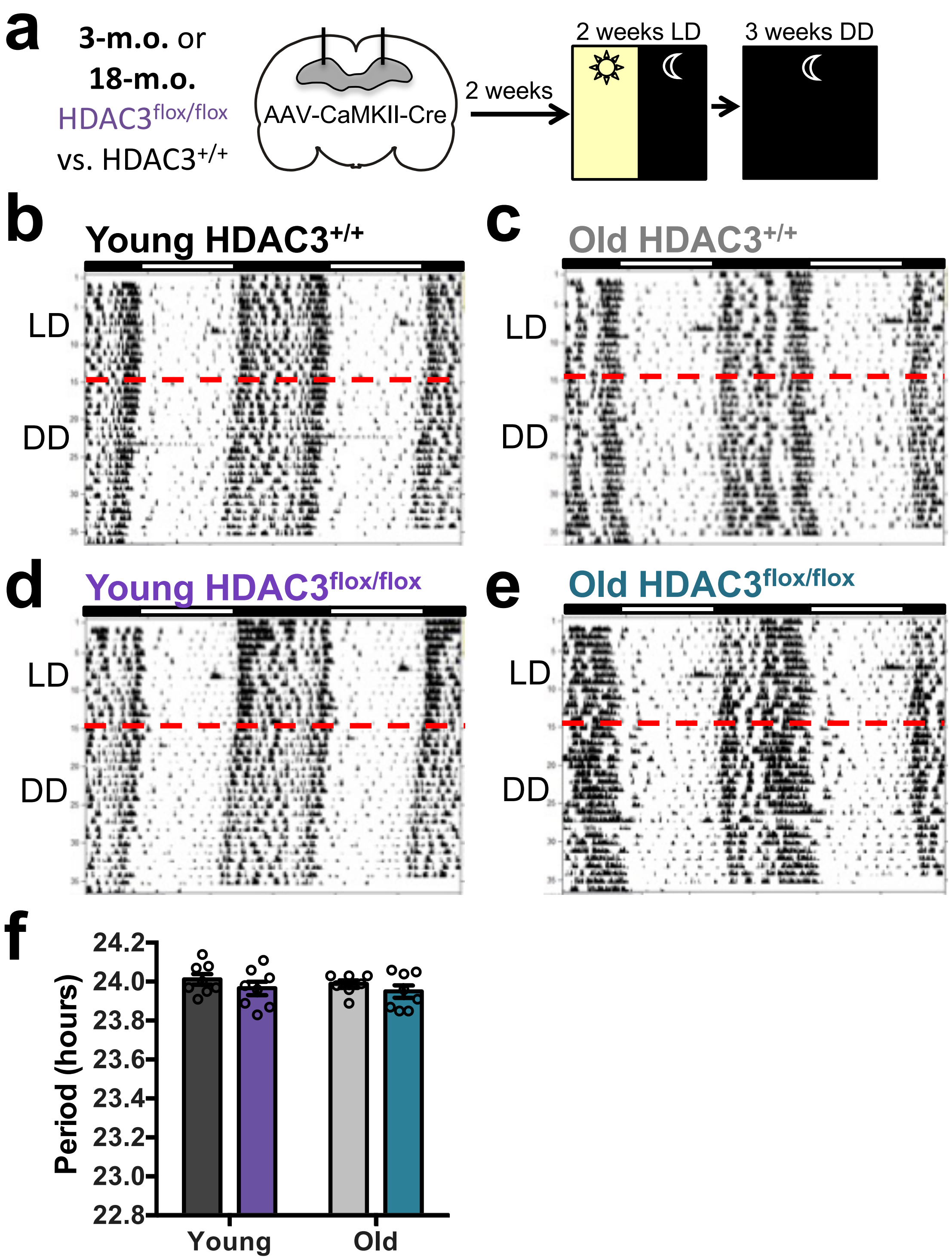

Supplementary Figure 4. Deletion of HDAC3 in the DH does not affect circadian rhythm of young or old mice. (a) Experimental procedure. **(b-e)** Representative actograms depicting circadian behavior for each group. Red dotted line indicates the switch from LD to DD. **(b)** Young HDAC3$^{+/+}$ actogram. **(c)** Old HDAC3$^{+/+}$ actogram. **(d)** Young HDAC3$^{flox/flox}$ actogram. **(e)** Old HDAC3$^{flox/flox}$ actogram. **(f)** HDAC3 deletion in DH did not affect free-running tau in either young or old mice (no significant effects, n=8,8,7,8; all males). Data are presented as mean ±SEM.

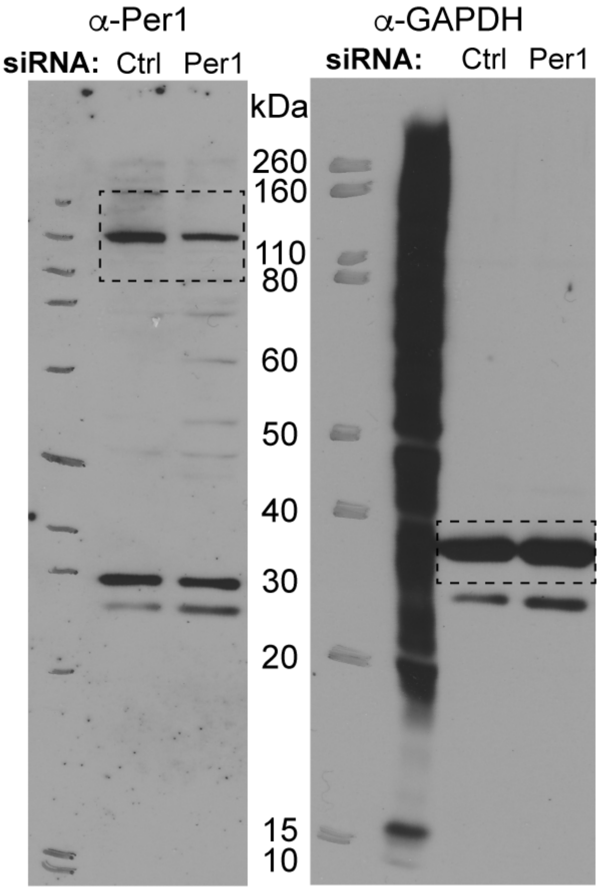

Supplementary Figure 5. Full-length western blot illustrating siRNA-mediated reduction in PER1. Proteins were identified with an anti-Per1 antibody (left, 30-minute exposure) followed by stripping and an anti-GAPDH antibody (right, 10-second exposure). Dotted boxes indicate the portion of the figure shown in Figure 4d.

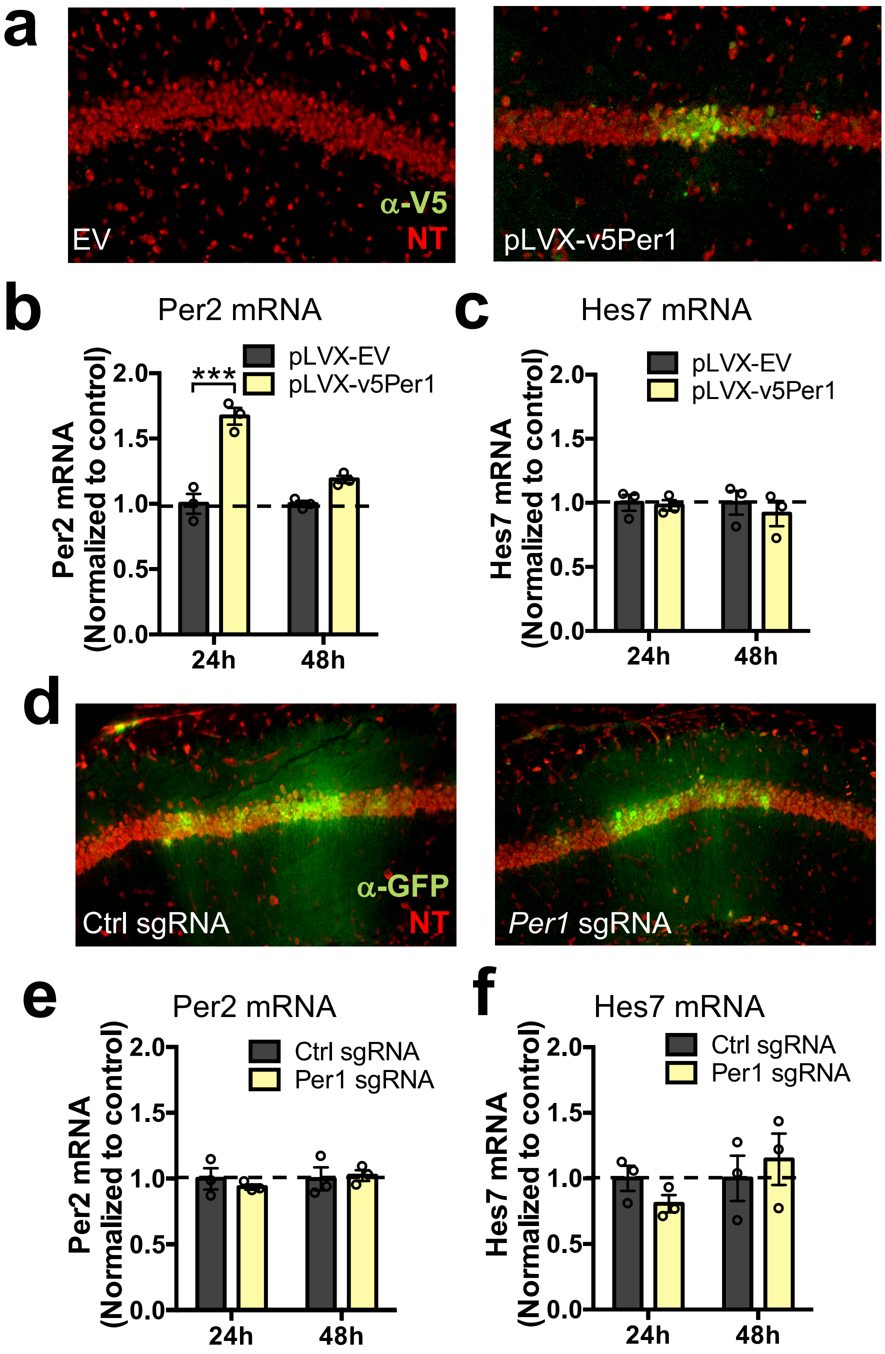

Supplementary Figure 6. Per1-pLVX and Per1 CRISPR-SAM expression in the dorsal hippocampus of aging mice. (a) Example immunofluorescence images of V5 (green) expression in mice injected with either pLVX-EV (left) or pLVX-v5Per1 (right). Cell bodies were counterstained with a fluorescent Nissl stain (NeuroTrace, red). **(b)** *Per2* mRNA was significantly increased 24h after transfection of pLVX-v5Per1 in HT22 cells compared to cells transfected with pLVX-EV (Two-way ANOVA, significant Group x Time interaction ($F(1,8)=21.28$, $p<0.01$), Sidak's *post hoc* tests, ***$p<0.001$, n=3,3,3,3). **(c)** *Hes7* mRNA was unchanged 24h or 48h after pLVX-v5Per1 transfection (Two-way ANOVA, no significant main effect or interaction, n=3,3,3,3). **(d)** Example immunofluorescence images of GFP (green) expression in mice injected with the CRISPR-SAM system either with sgRNA lacking the *Per1* target sequence (Ctrl sgRNA, left) or with the Per1 guide RNA (Per1 sgRNA, right). Both groups show expression of GFP, a component of the dCas9-VP64-GFP plasmid injected in both groups. **(e)** *Per2* mRNA was unchanged by CRISPR-SAM transfection (Two-way ANOVA, no significant main effect or interaction, n=3,3,3,3). **(f)** *Hes7* mRNA was also unchanged by *Per1* CRISPR-SAM transfection (Two-way ANOVA, no significant main effect or interaction, n=3,3,3,3). Data are presented as mean ±SEM.

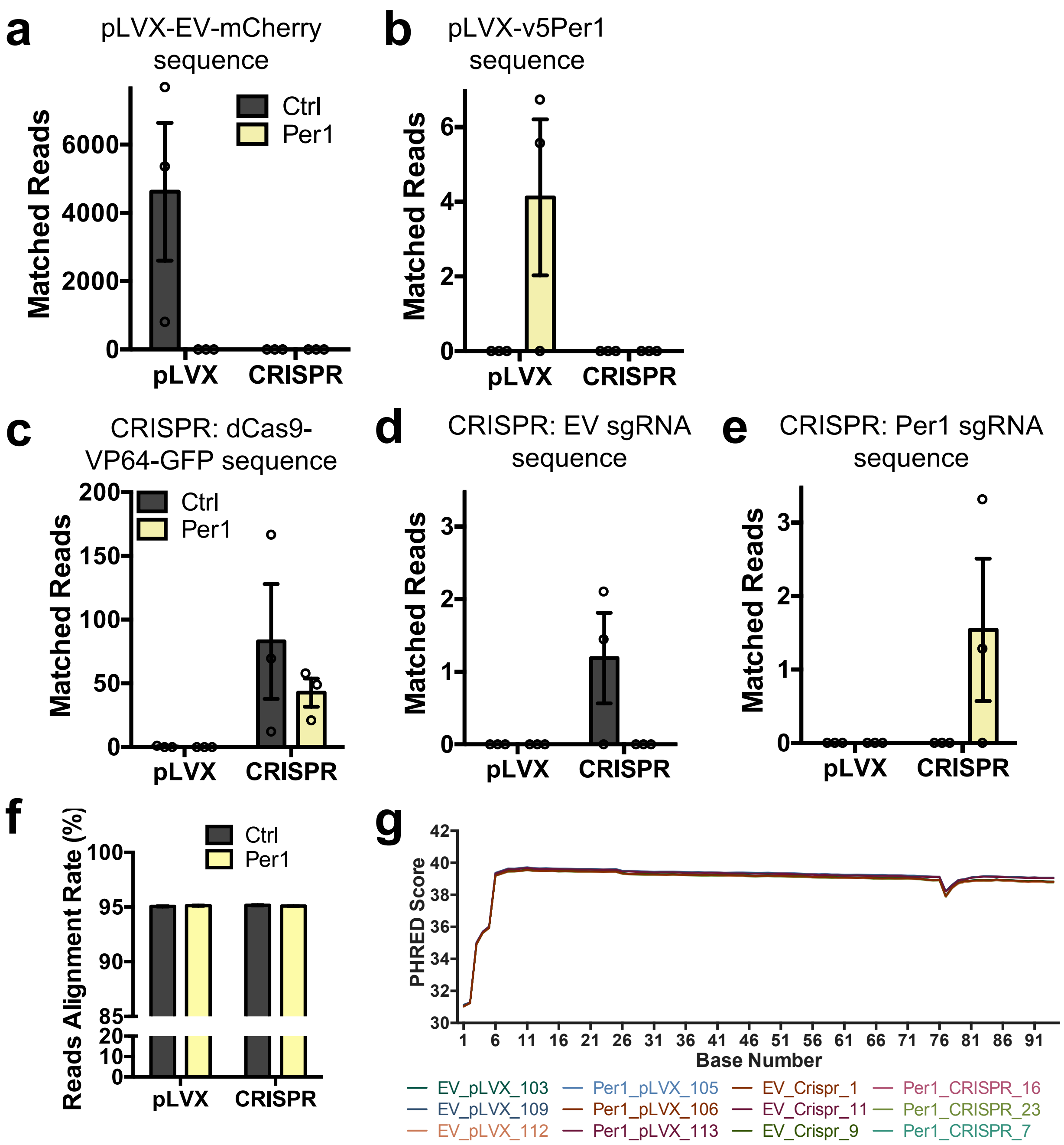

Supplementary Figure 7. Transcript detection for Per1-overexpression plasmids using RNA-sequencing. (a) The pLVX-EV-mCherry sequence was only detected in hippocampal samples from animals injected with pLVX-EV-mCherry **(b)** The pLVX-v5Per1 sequence was only detected in the group injected with pLVX-v5Per1. **(c)** The dCas9-VP64-GFP sequence was detected in both CRISPR-SAM groups (EV sgRNA and Per1 sgRNA) but not in either pLVX group. **(d)** The EV sgRNA sequence was only detected in the CRISPR-SAM group injected with EV sgRNA. **(e)** The Per1 sgRNA sequence was only detected in the CRISPR-SAM group injected with Per1 sgRNA. n=3/group. Ctrl = control virus (EV or ctrl sgRNA); Per1 = Per1 OE virus (pLVX-v5Per1 or Per1 sgRNA). Data are presented as mean ±SEM. **(f)** Percent reads alignment rate for each of the four groups was similar. **(g)** Mean PHRED quality scores for each animal.

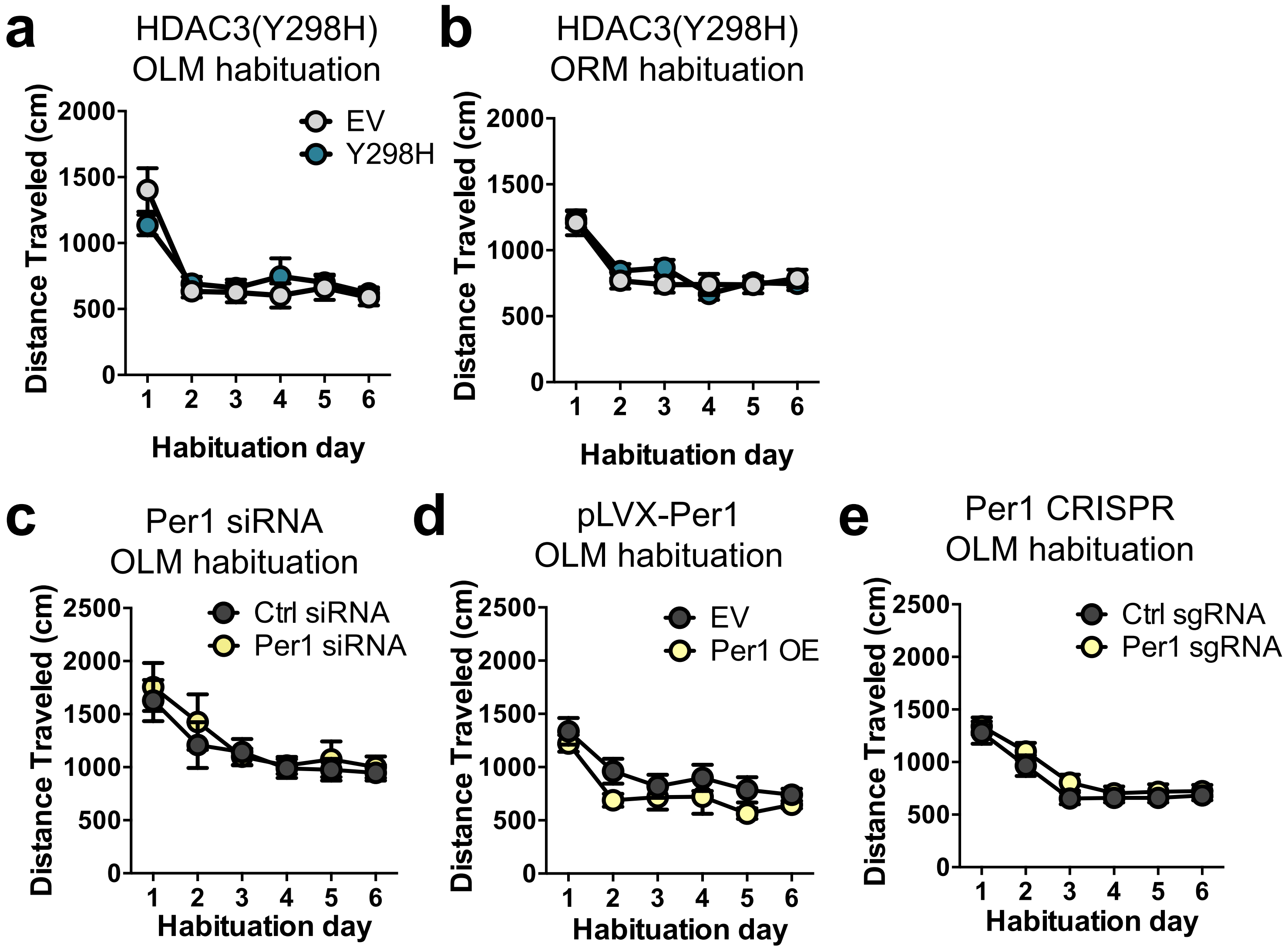

Supplementary Figure 8. All groups show normal movement during habituation. (A-E) Total distance traveled during each day of habituation for mice in figures 1D **(a)**, 1I **(b)**, 4E **(c)**, 5C **(d)**, and 5G **(e)**. No group differences were observed and individual comparisons between groups each day revealed no significant differences. Data are presented as mean ±SEM.

Increased expression					
	Gene	Homecage (Mean)	Trained (Mean)	Fold Change (log)	p-value
1	AF357426	0.123	1.062	0.939	9.75919E-12
2	A230065H16Rik	0.651	3.996	3.345	1.28698E-10
3	Glp1r	0.227	1.091	0.864	2.66057E-08
4	Prdm12	0.072	0.456	0.383	2.43083E-07
5	Gh	0.007	0.350	0.343	2.02023E-06
6	Ano2	0.389	1.077	0.688	3.13868E-06
7	Sln	0.668	2.403	1.734	3.40852E-06
8	Igfbpl1	1.597	2.459	0.862	5.53357E-06
9	Ctxn3	0.943	2.588	1.646	6.99663E-06
10	Ngb	0.552	1.313	0.761	8.55593E-06
11	Magel2	0.141	0.418	0.277	2.82686E-05
12	Mir702	0.000	0.110	0.110	3.61675E-05
13	Isl1	0.018	0.239	0.221	5.03269E-05
14	C130060K24Rik	0.051	0.230	0.179	7.6283E-05
15	Gpr88	0.762	2.072	1.310	0.000132281
16	Mir7009	0.000	0.158	0.158	0.000179688
17	Zic1	2.590	7.161	4.571	0.000236958
18	Sost	0.056	0.253	0.197	0.000278343
19	C130074G19Rik	5.293	8.409	3.116	0.000297396
20	Dlx1	4.424	6.604	2.180	0.000537434
Decreased expression					
	Gene	Homecage (Mean)	Trained (Mean)	Fold Change (log)	p-value
1	Mir804	0.380	0.065	-0.316	4.79591E-08
2	DQ267100	1.702	0.561	-1.141	5.03473E-06
3	Mir486	0.312	0.058	-0.254	7.96871E-06
4	Cpne7	47.046	38.314	-8.732	8.44389E-06
5	Mir1668	0.447	0.064	-0.383	1.00325E-05
6	2310003N18Rik	0.039	0.000	-0.039	2.98682E-05
7	D16Ertd519e	0.029	0.000	-0.029	0.000129378
8	Mir698	0.066	0.000	-0.066	0.000159265
9	1700020M21Rik	0.028	0.000	-0.028	0.000168869
10	Mir367	0.039	0.000	-0.039	0.000393218
11	Grp	8.839	6.777	-2.061	0.000451035
12	Eif3j1	0.445	0.000	-0.445	0.001197766
13	Snord123	0.136	0.000	-0.136	0.001221741
14	Hbq1a	0.295	0.068	-0.227	0.001656455
15	Mir202	0.029	0.000	-0.029	0.002539309
16	Nov	37.795	30.697	-7.097	0.002754578
17	Snora34	1.884	0.725	-1.160	0.002813874
18	Mir669n	0.090	0.000	-0.090	0.003749518
19	Mir6947	0.080	0.014	-0.065	0.004189413
20	Camp	0.057	0.000	-0.057	0.004231699

Supplementary Table 1

Genes with increased (top) or decreased (bottom) expression in young (3-m.o.) wildtype mice 60m after OLM training (trained) compared to baseline (homecage). The top 20 genes increased or decreased (based on *p*-value, homecage vs trained) are shown.

	Increased expression				
	Gene	Homecage (Mean)	Trained (Mean)	Fold Change (log)	p-value
1	Nr4a3	13.257	20.963	7.706	7.43057E-06
2	Ak4	6.212	9.271	3.059	8.98888E-05
3	Mir344d-2	0.000	0.249	0.249	0.000105067
4	Slit2	1.970	3.727	1.757	0.000166913
5	Tmem114	2.091	3.443	1.351	0.000181875
6	Mir7054	0.009	0.261	0.252	0.000278494
7	Sgk1	24.420	37.819	13.399	0.000317729
8	Mir7665	0.000	0.098	0.098	0.000451994
9	Mir7232	0.009	0.289	0.280	0.000463299
10	Gm11213	0.000	0.105	0.105	0.000771559
11	Sap18	1.153	2.875	1.722	0.000831838
12	Lpl	10.900	15.275	4.374	0.001252228
13	Cldn22	2.520	4.334	1.814	0.001502037
14	Cox8b	0.073	7.384	7.311	0.001810557
15	Pvrl3	6.285	9.501	3.216	0.001872061
16	Ccdc3	7.139	10.598	3.459	0.001908397
17	Coch	1.422	4.054	2.632	0.001920303
18	Arl4d	7.804	11.689	3.885	0.002276406
19	Mir7662	0.136	0.682	0.546	0.002427375
20	Pon1	0.000	0.509	0.509	0.002437193
	**Decreased expression**				
	**Gene**	**Homecage (Mean)**	**Trained (Mean)**	**Fold Change (log)**	**p-value**
1	Cd72	31.100	4.614	-26.487	9.05719E-07
2	Irf8	24.878	8.112	-16.766	1.64456E-06
3	Il4ra	10.307	2.185	-8.122	1.74389E-06
4	Fcgr4	28.396	5.438	-22.958	2.21299E-06
5	Themis2	9.935	2.454	-7.481	2.38824E-06
6	Snord43	0.445	0.000	-0.445	2.52343E-06
7	Klrb1b	1.652	0.115	-1.537	7.80601E-06
8	Ptprc	16.345	4.806	-11.539	7.83063E-06
9	Fcgr2b	29.445	8.814	-20.631	9.22626E-06
10	Scimp	4.059	0.557	-3.501	9.33703E-06
11	Srgn	47.757	13.503	-34.254	9.39187E-06
12	Ubd	8.176	0.417	-7.760	9.987E-06
13	Pik3ap1	16.416	6.053	-10.363	1.10568E-05
14	Tgm2	25.834	7.395	-18.439	1.12324E-05
15	Irf1	36.175	11.774	-24.400	1.13451E-05
16	Plac8	53.527	7.757	-45.770	1.17837E-05
17	Ccr5	12.634	4.444	-8.190	1.293E-05
18	Batf2	5.442	1.303	-4.139	1.58681E-05
19	Dusp2	4.600	1.214	-3.386	1.67166E-05
20	Fgl2	20.901	5.857	-15.044	1.68507E-05

Supplementary Table 2
Genes with increased (top) or decreased (bottom) expression in old (18-m.o.) wildtype mice 60m after OLM training (trained) compared to baseline (homecage). The top 20 genes increased or decreased (based on *p*-value, homecage vs trained) are shown.

Increased expression					
	**Gene**	**Homecage (Mean)**	**Trained (Mean)**	**Fold Change (log)**	**p-value**
1	Kcnc4	11.692	22.464	10.772	2.18475E-10
2	Plk5	10.516	21.224	10.708	3.79101E-07
3	Arl4d	8.143	12.922	4.780	2.34263E-06
4	Gabrd	11.412	18.535	7.123	2.60145E-06
5	Tnfrsf25	2.214	4.856	2.642	3.03957E-06
6	Mef2c	21.021	32.112	11.091	3.14128E-06
7	Sstr2	1.770	3.830	2.059	3.797E-06
8	Gfra2	6.925	11.530	4.605	4.6487E-06
9	Gsg1l	7.038	11.922	4.884	4.9045E-06
10	C1ql3	37.159	67.272	30.113	5.43282E-06
11	Grm2	12.253	23.605	11.351	7.13077E-06
12	Per1	13.384	19.212	5.828	8.99537E-06
13	Stk32c	32.308	48.977	16.669	1.62623E-05
14	Tmem132a	28.263	41.415	13.152	1.95184E-05
15	Pak6	8.027	12.580	4.553	2.09096E-05
16	Pcdh8	13.397	22.680	9.283	2.31057E-05
17	Hlf	21.758	32.986	11.228	2.37294E-05
18	Trank1	23.176	35.030	11.854	2.75255E-05
19	Nrep	10.592	18.779	8.187	3.05979E-05
20	Npy1r	6.282	10.844	4.562	3.98204E-05
**Decreased expression**					
	**Gene**	**Homecage (Mean)**	**Trained (Mean)**	**Fold Change (log)**	**p-value**
1	Cd180	22.518	6.494	-16.024	1.82021E-12
2	Cst7	51.825	16.930	-34.895	5.39092E-11
3	Cxcl13	49.009	10.270	-38.739	1.20531E-10
4	B2m	2050.156	822.502	-1227.654	4.097E-10
5	Cd36	2.865	0.396	-2.469	7.55399E-10
6	H2-DMa	36.402	13.806	-22.596	8.27047E-10
7	Hcar2	6.767	1.549	-5.218	1.25317E-09
8	Il18bp	33.348	8.993	-24.355	4.23861E-09
9	Rarres2	43.032	15.889	-27.143	4.6807E-09
10	H2-Oa	11.690	3.175	-8.515	5.08093E-09
11	Psmb9	81.679	27.668	-54.011	5.723E-09
12	Gdf3	2.085	0.467	-1.619	6.91066E-09
13	Slamf8	9.245	2.511	-6.734	7.06283E-09
14	Bin2	18.134	8.050	-10.084	7.31527E-09
15	H2-Q4	84.563	30.819	-53.744	7.36118E-09
16	H2-DMb1	19.944	6.815	-13.128	1.01291E-08
17	H2-L	61.942	23.548	-38.394	1.19768E-08
18	Gpr84	8.355	3.449	-4.906	1.58238E-08
19	Lair1	12.122	5.280	-6.842	1.83705E-08
20	Ly86	100.153	45.634	-54.519	2.08906E-08

Supplementary Table 3

Genes with increased (top) or decreased (bottom) expression in old (18-m.o.) HDAC3$^{flox/flox}$ mice 60m after OLM training (trained) compared to baseline (homecage). The top 20 genes increased or decreased (based on *p*-value, homecage vs trained) are shown.

Quantitative real-time PCR Primers and Probes			
**Gene**	**Forward (5' to 3')**	**Reverse (5' to 3')**	**Probe**
GAPDH	ATGGTGAAGGTCGGTGTGA	AATCTCCACTTTGCCACTGC	TGGCGGTATTGG
Per1	TGTCCGTCACCAGTCAGTGT	CCAGGCAGGTCTTCCATC	#22 (CTCCACCA)
Per2	TCCGAGTATATCGTGAAGAACG	CAGGATCTTCCCAGAAACCA	#5 (TGTGGCTG)
Hes7	GAGAGGACCAGGGACCAGA	TTCGCTCCCTCAAGTAGCC	#78 (AGCTGGAG)
NR4A1	AGCTTGGGTGTTGATGTTCC	GCCCCTGCCTGGTAGAGT	#93 (TCTGGTCC)
NR4A2	TTGCAGAATATGAACATCGACA	GTTCCTTGAGCCCGTGTCT	#2 (TTCTCCTG)
Arc	CGGGACCTGTACCAGACACT	GCCCACCACATACTGAATGA	#63 (AGGAGGAG)
cFos	GGGGCAAAGTAGAGCAGCTA	AGCTCCCTCCTCCGATTC	#46 (ATGGCTGC)
Hdac3	TTCAACGTGGGTGATGACTG	TTAGCTGTGTTGCTCCTTGC	#32 (CTGCTCCC)
Hdac3-V5	TGGAGATTCTCGAGGGTAAGC	ATGCCACCCGTAGATCTGG	#91 (CTCTCCTC)

ChIP Primers		
**Gene**	**Forward (5' to 3')**	**Reverse (5' to 3')**
Per1 CRE	CAGCTGCCTCGCCCCGCCTC	CCCAAGCAGCCATTGCTCGC
Per1 E-Box	CCCTCACTTCCCTTTCATTATTGACG	TGCATAATGCCAGGCCCTGCCCCTCATTGG
Per1 Prox	ACTGGGTGTAGCCAGCAGAT	ACAGTGGAGGACGAAACAGG
Arc	CGGGACCTGTACCAGACACT	GCCCACCACATACTGAATGA
cFos	TACGACCCCTTCAGGCATAC	GTTTTAAAGGACGGCAGCAC

Supplementary Table 4
List of quantitative real-time PCR primers and probes (Roche Universal Probe Library) and ChIP primers (designed with Primer 3).
